# Genome sequence and methylome profile of *Natrinema pallidum* strain NMX15-1, a halophilic archaeon, from Permian Period halite, Carlsbad, New Mexico

**DOI:** 10.1128/mra.01287-24

**Published:** 2025-05-20

**Authors:** Aram Khoshnaw, Beatriz Toledo Akiti, Tamas Vincze, Priya DasSarma, Alexey Fomenkov, Dennis W. Powers, Richard J. Roberts, Shiladitya DasSarma

**Affiliations:** 1 Blue Marble Space Institute of Science, Seattle, Washington, USA; 2 New England Biolabs, Ipswich, Massachusetts, USA; 3 Department of Microbiology and Immunology, School of Medicine, University of Maryland, Baltimore, Maryland, USA; 4 University of Mississippi 8083, University, Mississippi, USA; 5 Institute of Marine and Environmental Technology, University System of Maryland 1069, Baltimore, Maryland, USA; Portland State University, Portland, Oregon, USA

**Keywords:** Haloarchaea, halophilic archaea, acidic proteome, DNA methylation, core Haloarchaeal Orthologous Groups (cHOGs), replication proteins, microbial rhodopsins, photolyase

## Abstract

We report the 3.8-Mbp genome sequence of an extreme Haloarchaeon, *Natrinema pallidum* strain NMX15-1, isolated from 250-million-year-old halite samples of the Permian Period. PacBio’s long-read sequencing revealed a genome comprising a chromosome (3.5-Mbp) and three plasmids (203, 71, and 56-Kbp). The proteome is highly acidic and includes 3,784 proteins.

## ANNOUNCEMENT

Halophilic Archaea (Haloarchaea) are interesting organisms for biotechnology applications, evolutionary studies, and as models for astrobiology research (1). *Natrinema pallidum* strain NMX15-1 (NMX15-1) was isolated from 250-million-year-old halite samples of the Permian Period (2, 3). Sampling was conducted at a depth of 655 meters below surface level (382 meters above mean sea level) at an ambient temperature of 26°C in the Salado Formation near Carlsbad, New Mexico (32°22′18″N, 103°47′37″W) on 6 November 2019 (4).

Surface-sterilized halite samples were enriched in CM^+^ media, followed by incubation at 37°C with shaking (220 rpm) and illumination for 2 months (New Brunswick Innova 4230; New Brunswick, NJ, USA) (5, 6). Enriched cultures were plated on CM^+^ agar, and candidate colonies were repeatedly cultured until colonies appeared uniform through three rounds of subculturing (6, 7).

A Monarch Genomic DNA Purification Kit T3010S (NEB) was used for DNA extraction from a liquid culture grown in CM^+^ medium, and Single-Molecule Real-Time (SMRT) sequencing was performed on the Sequel II platform (Pacific Biosciences, CA) (8). Briefly, DNA samples (2 µg) were sheared to an average size of ~10 kb, and DNA libraries were prepared using a SMRTbell Express Template Prep Kit 2.0. Qualification and quantification of the SMRTbell libraries were made on a Qubit fluorometer (Invitrogen, OR) and a 2100 Bioanalyzer (Agilent Technologies, CA). Subsequently, the ~10 kb SMRT library was barcoded and sequenced with a 30 h collection time. The 188,124 HiFi subreads (7,103 bp mean read length, and 8,180 bp N_50_ read length) yielding 1.352 Gb of high-quality sequencing data were collected and *de novo* assembled using the Microbial Analysis pipeline (SMRT Link v11.1.0.166339, with automatic quality control of HiFi reads and kinetic analysis of modified motifs). The polished assemblies generated four closed circular contigs, a 3.5-Mbp chromosome, and three plasmids (203, 71, and 56-Kbp), which were manually rotated to match homologous replicons in GenBank for *Natrinema pallidum* BOL6-1 (Table 1) (5). SMRT sequencing also detected DNA methylation sites in the genome (9).

**TABLE 1:** *Natrinema pallidum* strain NMX15-1 genome properties

Replicon	Size (bp)	GC (%)	Coverage	Gene no.	GenBank
Chromosome	3,503,931	64.5	351	3,537	CP171781
pNMX15-1_203	203,204	60.5	301	187	CP171782
pNMX15-1_71	70,938	60.5	504	113	CP171784
pNMX15-1_56	56,435	61.0	446	83	CP171783
Whole genome	3,834,508	64.3	353	3,920	Assembly

Taxonomy was determined by the National Center for Biotechnology Information (NCBI), and annotation was performed by NCBI’s Prokaryotic Genome Annotation Pipeline build 3190 (10, 11). The genome encodes 3,920 predicted genes, including 3 rRNA operons and 51 tRNA genes, 5 of which contain introns (Gly, Gln, Trp, Met, and Ile2) based on tRNAscan-SE 2.0 (12). Using IPC 2.0, a highly acidic proteome was detected, with a mean isoelectric point (pI) value of 4.7, a characteristic of Haloarchaea (13, 14).

Nearly all of the core Haloarchaeal Orthologous Groups (797) were found based on HaloWeb, EMBOSS 6.6.0.0, and BLAST 2.11.0 analysis (15–18). The genome is predicted to encode 19 Orc/Cdc6 proteins (Orc1, 4–7, 9, and 10), a single TATA-box-binding protein (TbpE) and 10 transcription initiation factor IIB proteins (TfbA, F, and G), as well as 11 Gvp proteins, bacteriorhodopsin (Bop), halorhodopsin (Hop), and photolyase (Phr2), and haloarchaeal insertion sequence (ISH) elements, ISH8, 11, and 12 (19–23).

Based on SMRT Link’s Base Modification and Motif Analysis application, three methylated DNA motifs are present in NMX15-1 and were recorded in the REBASE database (28). One is an asymmetrical m6A CATTC motif (M.NpaNMX151I, ACED83_04090), a second one, which is commonly found in Haloarchaea, is an m4C CTAG motif (M.NpaNMX151III, ACED83_01945), and the third is an m5C GACGTC motif (M.NpaNMX151II, ACED83_07330) (24, 25).

## Data Availability

The NMX15-1 genome (Bioproject: PRJNA412908, BioSample: SAMN43289003) has been deposited in GenBank under the following accession numbers: chromosome (CP171781), pNMX15-1_203 (CP171782), pNMX15-1_71 (CP171784), and pNMX15-1_56 (CP171783). Raw data are available in the NCBI Sequence Read Archive (SRR31014709).
